# ACCORD (ACcurate COnsensus Reporting Document): A reporting guideline for consensus methods in biomedicine developed via a modified Delphi

**DOI:** 10.1371/journal.pmed.1004326

**Published:** 2024-01-23

**Authors:** William T. Gattrell, Patricia Logullo, Esther J. van Zuuren, Amy Price, Ellen L. Hughes, Paul Blazey, Christopher C. Winchester, David Tovey, Keith Goldman, Amrit Pali Hungin, Niall Harrison

**Affiliations:** 1 Bristol Myers Squibb, Uxbridge, United Kingdom; 2 Centre for Statistics in Medicine, University of Oxford, and EQUATOR Network UK Centre, Oxford, United Kingdom; 3 Leiden University Medical Centre, Leiden, Netherlands; 4 Stanford Anesthesia, Informatics and Media Lab, Stanford University School of Medicine, Stanford, California, United States of America; 5 Camino Communications Ltd, Mansfield, United Kingdom; 6 Department of Medicine, University of British Columbia, Vancouver, British Columbia, Canada; 7 Oxford PharmaGenesis, Oxford, United Kingdom; 8 Green Templeton College, University of Oxford, Oxford, United Kingdom; 9 Journal of Clinical Epidemiology, London, United Kingdom; 10 Global Medical Affairs, AbbVie, North Chicago, Illinois, United States of America; 11 Faculty of Medical Sciences, Newcastle University, Newcastle, United Kingdom; 12 OPEN Health Communications, Marlow, United Kingdom

## Abstract

**Background:**

In biomedical research, it is often desirable to seek consensus among individuals who have differing perspectives and experience. This is important when evidence is emerging, inconsistent, limited, or absent. Even when research evidence is abundant, clinical recommendations, policy decisions, and priority-setting may still require agreement from multiple, sometimes ideologically opposed parties. Despite their prominence and influence on key decisions, consensus methods are often poorly reported. Our aim was to develop the first reporting guideline dedicated to and applicable to all consensus methods used in biomedical research regardless of the objective of the consensus process, called ACCORD (ACcurate COnsensus Reporting Document).

**Methods and findings:**

We followed methodology recommended by the EQUATOR Network for the development of reporting guidelines: a systematic review was followed by a Delphi process and meetings to finalize the ACCORD checklist. The preliminary checklist was drawn from the systematic review of existing literature on the quality of reporting of consensus methods and suggestions from the Steering Committee. A Delphi panel (*n* = 72) was recruited with representation from 6 continents and a broad range of experience, including clinical, research, policy, and patient perspectives. The 3 rounds of the Delphi process were completed by 58, 54, and 51 panelists. The preliminary checklist of 56 items was refined to a final checklist of 35 items relating to the article title (*n* = 1), introduction (*n* = 3), methods (*n* = 21), results (*n* = 5), discussion (*n* = 2), and other information (*n* = 3).

**Conclusions:**

The ACCORD checklist is the first reporting guideline applicable to all consensus-based studies. It will support authors in writing accurate, detailed manuscripts, thereby improving the completeness and transparency of reporting and providing readers with clarity regarding the methods used to reach agreement. Furthermore, the checklist will make the rigor of the consensus methods used to guide the recommendations clear for readers. Reporting consensus studies with greater clarity and transparency may enhance trust in the recommendations made by consensus panels.

## Abstract 2

Gattrell and colleagues developed the ACCORD reporting guideline to guide the reporting of biomedical studies using consensus methods.

## Background

Evidence-based medicine relies on (1) the best available evidence; (2) patients’ values, preferences, and knowledge; and (3) healthcare professionals’ experience and expertise [1,2]. When healthcare professionals need to make clinical decisions, or when recommendations or guidance are needed and there is uncertainty on the best course of action, such as when evidence is emergent, inconsistent, limited, or absent—not least in rapidly evolving fields such as pandemics [3]—the collation and dissemination of knowledge, experience, and expertise becomes critical. Coordinating this process may be best achieved through the use of formal consensus methods [4] such as those described in Table 1.

**Table 1:** A selection of common consensus methods used in healthcare-related activities or research.

Method	Characteristics
Delphi [5,6]	• Anonymity • Iteration over multiple rounds of voting • Feedback after each round
Nominal group technique [7]	A face-to-face group interaction comprising 4 stages: • Solo idea generation • Round-robin feedback of ideas • Clarification of ideas through discussion • Voting to prioritize or rank ideas
RAND/UCLA appropriateness method (RAM) [8]	A method developed to combine the best available scientific evidence with the collective judgment of experts to yield a statement regarding, for example, the appropriateness of performing a procedure. Stages include: • Literature review • Development of statements • Expert scoring of statements
Consensus meetings [9]	Simple meetings with discussion to reach consensus, including voting in structured or more informal formats

UCLA, University of California, Los Angeles

Consensus methods are widely applied in healthcare (Table 2). However, the specific method has the potential to affect the result of a consensus exercise and shape the recommendations generated. In addition, the expertise needed to contribute to the consensus process will vary depending on the research subject, and a range of participants may be required, including, but not limited to, clinical guideline developers, clinical researchers, healthcare professionals, epidemiologists, ethicists, funders, journal editors, laboratory specialists, medical publication professionals, meta-researchers, methodologists, pathologists, patients and carers/families, pharmaceutical companies, public health specialists, policymakers, politicians, research scientists, surgeons, systematic reviewers, and technicians.

**Table 2:** Examples of applications of consensus methods in healthcare-related research.

Study purpose	How consensus helps
Clinical practice guidelines [10]	Translating evidence into clinical recommendations, particularly where the evidence is uncertain, and incorporating multiple perspectives; creating clinical recommendations based on experience
Diagnostic guidelines [11]	Defining the markers, signs, and symptoms or thresholds that indicate a specific condition
Disease classification [11]	Classifying disease type or severity
Establishing research priorities [12]	Defining and ranking priorities in the context of limited resources
Developing core outcome sets [13]	Defining the most important and clinically significant outcomes in research
Formulating policy [14]	Analyzing and interpreting evidence, its biases and strengths, to inform policies. People gathered in consensus activities can analyze evidence together from different perspectives
Reporting guidelines [15]	Developing guidance on what should be reported in scientific articles to enhance transparency and methodological rigor

Consensus obtained from a group of experts using formal methods is recognized as being more reliable than individual opinions and experiences [16–18]. Consensus methods help to overcome the challenges of gathering opinions from a group, such as discussions being dominated by a small number of individuals, peer pressure to conform to a particular opinion, or the risk of group biases affecting overall decision-making [4].

Despite their critical role in healthcare and policy decision-making, consensus methods are often poorly reported [19]. Generic problems include inconsistency and lack of transparency in reporting, as well as more specific criticisms such as lack of detail regarding how participants or steering committee members were selected, missing panelist background information, no definition of consensus, missing response rates after each consensus round, no description of level of anonymity or how anonymity was maintained, and a lack of clarity over what feedback was provided between rounds [19].

Reporting guidelines can enhance the reporting quality of research [20–22], and the absence of a universal reporting guideline for studies using consensus methods may contribute to their well-documented suboptimal reporting quality [5,19,23–25]. A systematic review found that the quality of reporting of consensus methods in health research was deficient [19], and a methodological review found that articles that provided guidance on reporting Delphi methods vary widely in their criteria and level of detail [25]. The Conducting and Reporting Delphi Studies (CREDES) guideline was designed to support the conduct and reporting of Delphi studies, with a focus on palliative care [26]. The 23-item AGREE-II instrument [27], which is widely used for reporting clinical practice guidelines, and COS-STAR for reporting core outcome set development [28], both contain a very limited number of items related to consensus.

Therefore, a comprehensive guideline is needed to report the numerous methods available to assess and/or guide consensus in medical research. The ACcurate COnsensus Reporting Document (ACCORD) reporting guideline project was initiated to fulfill this need. We followed EQUATOR Network–recommended best practices for reporting guideline development, which included a systematic review and consensus exercise. Our aim was to develop a new tool, applicable worldwide, that will facilitate the rigorous and transparent reporting of all types of consensus methods across the spectrum of health research [29]. A comprehensive reporting guideline will enable readers to understand the consensus methods used to develop recommendations and therefore has the potential to positively impact patient outcomes.

## Methods

### Scope of ACCORD

ACCORD is a meta-research project to develop a reporting guideline for consensus methods used in health-related activities or research (Table 2) [29]. The guideline was designed to be applicable to simple and less structured methods (such as consensus meetings), more systematic methods (such as nominal group technique or Delphi), or any combination of methods utilized to achieve consensus. Therefore, the ACCORD checklist should be applicable to work involving any consensus methods. In addition, although ACCORD has been structured to help reporting in a scientific manuscript (with the traditional article sections such as introduction, methods, results, and discussion), the checklist items can assist authors in writing other types of text describing consensus activities.

ACCORD is a reporting guideline that provides a checklist of items that we recommend are included in any scientific publication in healthcare reporting the results of a consensus exercise. However, it is not a methodological guideline. It is not intended to provide guidance on how researchers and specialists should design their consensus activities, and it makes no judgment on which method is most appropriate in a particular context. Furthermore, ACCORD is not intended to be used for reporting research in fields outside health, such as social sciences, economics, or marketing.

### Study design, setting, and ethics

The ACCORD project was registered prospectively on January 20, 2022 on the Open Science Framework [30] and the EQUATOR Network website [31], and received ethics approval from the Central University Research Ethics Committee at the University of Oxford (reference number: R81767/RE001). The ACCORD protocol has been previously published [29] and followed the EQUATOR Network recommendations for developing a reporting guideline [32,33], starting with a systematic review of the literature [19], followed by a modified Delphi process. In a planned change to the Delphi method as originally formulated, the preliminary list for voting was based on the findings of this systematic review rather than initial ideas or statements from the ACCORD Delphi panel, although the panel could suggest items during the first round of voting. In addition, the ACCORD Steering Committee made final decisions on item inclusion and refined the checklist wording as described below.

### ACCORD Steering Committee

WTG and NH founded the ACCORD project, seeking endorsement from the International Society of Medical Publication Professionals (ISMPP) in April 2021. ISMPP provided practical support and guidance on the overall process at project outset but was not involved in checklist development. The ACCORD Steering Committee, established over the following months, was multidisciplinary in nature and comprised researchers from different countries and settings. Steering Committee recruitment was iterative, with new members invited as needs were identified by the founders and existing committee, to ensure inclusion of the desired range of expertise or experience. Potential members were identified via ISMPP, literature research, professional connections, and network recommendations. When the protocol was submitted for publication, the Steering Committee had 11 members (WTG, PL, EJvZ, AP, CCW, DT, KG, APH, NH, and Robert Matheis [RM] from ISMPP). Bernd Arents joined the Steering Committee in July 2021 but left in December of that year, as did RM in August 2022, both citing an excess of commitments as their reason for stepping down. Patient partners were invited as Delphi panelists. Paul Blazey joined the Steering Committee in September 2022 as a methodologist to support the execution of the ACCORD Delphi process and provide additional expertise on consensus methods.

The final Steering Committee responsible for the Delphi process and development of the checklist had members working in 4 different countries: Canada, United Kingdom, United States of America, and the Netherlands. A wide range of professional roles was represented by the Steering Committee with several members bringing experience from more than one area including clinician practitioners (medical doctor, physical therapist), methodologists (consensus methodologist, research methodologist, expert in evidence synthesis), medical publication professionals (including those working in the pharmaceutical industry), journal editors, a representative of the EQUATOR Network, and a representative of the public (S1 Text).

### Protocol development

The ACCORD protocol was developed by the Steering Committee before the literature searches or Delphi rounds were commenced and has been published previously [29]. An overview of the methods used, together with some amendments made to the protocol during the development of ACCORD in response to new insights, is provided below.

### Systematic review and development of preliminary checklist

A subgroup of the Steering Committee conducted a systematic review with the dual purpose of identifying existing evidence on the quality of reporting of consensus methods and generating the preliminary draft checklist of items that should be reported [19]. The systematic review has been published [19] and identified 18 studies that addressed the quality of reporting of consensus methods, with 14 studies focused on Delphi only and 4 studies including Delphi and other methods [19]. A list of deficiencies in consensus reporting was compiled based on the findings of the systematic review. Items in the preliminary checklist were subsequently derived from the systematic review both from the data extraction list (*n* = 30) [19] and from other information that was relevant for reporting consensus methods (*n* = 26) [19].

Next, the Steering Committee voted on whether the preliminary checklist items (*n* = 56) should be included in the Delphi via 2 anonymous online surveys conducted using Microsoft Forms (See S2 Text). There were 5 voting options: “Strongly disagree,” “Disagree,” “Agree,” “Strongly agree,” and “Abstain/Unable to answer.” NH processed the results in Excel, and WTG provided feedback and therefore neither voted. Items that received sufficient support (i.e., >80% of respondents voted “Agree”/“Strongly agree”) were included in the Delphi, while the rest were discussed by the Steering Committee for potential inclusion or removal. During the first survey, Steering Committee members could propose additional items based on their knowledge and expertise. These new items were voted on in the second Steering Committee survey. Upon completion of this process, the Steering Committee approved and updated the preliminary draft checklist, which was then prepared for voting on by the Delphi panel. Items were clustered or separated as necessary for clarity.

### Delphi panel composition

Using an anonymous survey (June 9–13, 2022), the Steering Committee voted on the desired profile of Delphi panelists for the ACCORD project. There was unanimous agreement that geographic representation was important, and the aim was to recruit from all continents (thereby covering both Northern and Southern hemispheres) and include participants from low-, middle-, and high-income countries to account for potential differences in cultural and ideological ways of reaching agreement. The aim was to include a broad range of participants: clinicians, researchers experienced in the use of consensus methods and in clinical practice guideline development, patient advocates, journal editors, publication professionals and publishers, regulatory specialists, public health policymakers, and pharmaceutical company representatives. As described in the ACCORD protocol [29], there are no generally agreed standards for the panel size in Delphi studies, although panels of 20 to 30 are common. The target panel size (approximately 40 panelists) was therefore guided by the desired representation and to ensure an acceptable number of responses (20, assuming a participation rate of 50%) in the event of withdrawals or partial completion of review.

### Delphi panel recruitment

Potential participants for the Delphi panel were identified in several ways: from the author lists of publications included in the systematic review, from invitations circulated via an EQUATOR Network newsletter (October 2021) [34] and at the European Meeting of ISMPP in January 2022, and by contacting groups potentially impacted by ACCORD (e.g., the UK National Institute for Health and Care Excellence [NICE]). Individuals were also invited to take part through the ACCORD protocol publication [29], and the members of the Steering Committee contacted individuals in their networks to fill gaps in geographical or professional representation. To minimize potential bias, none of the Steering Committee participated in the Delphi panel.

Invitations were issued to candidate panelists who satisfied the inclusion criteria. While participants were not generally asked to suggest other panel members, in some cases, invitees proposed a colleague to replace them on the panel. Only the Steering Committee members responsible for administering the Delphi had access to the full list of ACCORD Delphi panel members. Panelists were invited by email, and reminder emails were sent to those who did not respond. Out of the 133 panelists invited, 72 agreed to participate. No panelists or Steering Committee members were reimbursed or remunerated for taking part in the ACCORD project.

### Planned Delphi process

The Delphi method was chosen to validate the checklist, in line with recommendations for developing reporting guidelines [32]. A 3-round Delphi was planned to allow for iteration, with the option to include additional rounds if necessary. Panelists who agreed to take part received an information pack containing an introductory letter, a plain language summary, an informed consent statement, links to the published protocol and systematic review, and the items excluded by the Steering Committee (see S3 Text). Survey materials were developed by PL and PB in English and piloted by WTG and NH. Editorial and formatting changes were made following the pilot stage to optimize the ease of use of the survey. In an amendment to the protocol, the order of candidate items was not randomized within each manuscript section. The Jisc Online Survey platform (Jisc Services, Bristol, United Kingdom) was used to administer all Delphi surveys, ensuring anonymity through automatic coding of participants. Panelists were sent reminders to complete the survey via the survey platform, and one email reminder was sent to panelists the day before the deadline for each round.

The Delphi voting was modified to offer 5 voting options: “Strongly disagree,” “Disagree,” “Neither agree nor disagree,” “Agree,” and “Strongly agree.” Votes of “Neither agree nor disagree” were included in the denominator. The consensus threshold was defined a priori as ≥80% of a minimum of 20 respondents voting “Agree” or “Strongly agree.” Reaching the consensus threshold was not a stopping criterion. For inclusion in the final checklist, each item was required to achieve the consensus criteria following at least 2 rounds of voting. This ensured that all items had the opportunity for iteration between rounds (a central tenet of the Delphi method) [6] and enabled panelists to reconsider their voting position in light of feedback from the previous round.

In Round 1, panelists had the opportunity, anonymously, to suggest new items to be voted on in subsequent rounds. Panelists were also able to provide anonymous free-text comments in each round to add rationale for their chosen vote or suggest alterations to the item text. After each voting round, the comments were evaluated and integrated by WTG, PL, PB, and NH and validated by the Steering Committee. If necessary, semantic changes were made to items to improve clarity and concision.

Feedback given to participants between rounds included the anonymized total votes and the percentage in each category (see example in S4 Text) to allow panelists to assess their position in comparison with the rest of the group, as well as the relevant free-text comments on each item. Items that did not achieve consensus in Rounds 1 and 2 were revised or excluded based on the feedback received from the panelists. Items that were materially altered (to change their original meaning) were considered a new item. All wording changes were recorded. Panelists received a table highlighting wording changes as part of the feedback process so that they could see modifications to checklist items (for example feedback documents, see S5 Text).

Items reaching consensus over 2 rounds were removed from the Delphi for inclusion in the checklist. Items achieving agreement in Round 1, which then fell into disagreement in Round 2 were considered to have “unstable” agreement. These unstable items were revised based on qualitative feedback from the panel and were included for revoting in Round 3.

### Steering Committee checklist finalization process

Consistent with the protocol [29], following completion of the Delphi process, the Steering Committee was convened for a series of three 2-hour virtual workshops (March 7, 14, and 16, 2023) to make decisions and finalise the checklist. For each item, WTG, PL, PB, and NH presented a summary of voting, comments received, and a recommended approach. The possible recommended approaches are shown in S6 Text.

All recommendations (for example, to keep approved items, confirm exclusion of rejected items, etc.) were followed by an explanation of why WTG, PL, PB, or NH felt this would be the most appropriate action and a discussion between Steering Committee members in which the suggested action could be challenged and changed.

Grammatical changes were also considered at this stage but only where they did not change the meaning of an approved item. Following review of all items, the order of the checklist items was evaluated by WTG, PL, PB, and NH.

### Standardized terminology

After the consensus meetings, NH updated and standardized the terminology according to the type of information requested in the item to ensure consistency between items, and this was approved by the Steering Committee. This standardization of terminology incorporated rules established for the use of terms common in reporting guidelines, as shown in S7 Text, such as the difference between using “state” or “describe.” All but 2 items (R5 and O1) contain a verb from S7 Text.

## Results

### Delphi panel demographics

The Delphi panel included a diverse group of panelists, representing a wide range of geographical areas and professions (Table 3). Of the 72 participants who indicated their willingness to participate in the Delphi panel, 58 (81%) completed Round 1 and were invited to Round 2. Fifty-four participants completed Round 2 and were invited to Round 3, which was completed by 51 participants.

**Table 3:** Self-identified demographics of the Delphi panelists, per voting round.

Characteristic	Round 1 (*n* = 58) October 21–November 4, 2022	Round 2 (*n* = 54) December 21, 2022–January 16, 2023	Round 3 (*n* = 51) February 10–27, 2023
Gender, n (%)			
Female Male Nonbinary Prefer not to say	31 (53.4) 27 (46.6) 0 0	28 (51.9) 25 (46.3) 1 (1.9) 0	28 (54.9) 22 (43.1) 0 1 (2.0)
Geographic location of current primary residence and work, n (%)			
Africa Asia Europe North America Oceania South America	3 (5.2) 4 (6.9) 31 (53.4) 16 (27.6) 1 (1.7) 3 (5.2)	3 (5.6) 4 (7.4) 28 (51.9) 15 (27.8) 1 (1.9) 3 (5.6)	2 (3.9) 4 (7.8) 26 (51.0) 15 (29.4) 1 (2.0) 3 (5.9)
Background*, n (%)			
Clinician Journal editor Patient partner^†^ Policymaker Publications professional Researcher Other^‡^	16 (27.6) 8 (13.8) 6 (10.3) 3 (5.2) 17 (29.3) 29 (50.0) 11 (19.0)	14 (25.9) 6 (11.1) 6 (11.1) 3 (5.6) 17 (31.5) 29 (53.7) 6 (11.1)	13 (25.5) 8 (15.7) 5 (9.8) 4 (7.8) 15 (29.4) 24 (47.1) 8 (15.7)

*Panelists could select more than one option.

^†^In Rounds 2 and 3, this category was changed to Patient, Patient Partner, Family Member, or Carer.

^‡^Other occupation categories included:

In Round 1: Patient and Research Community: Pharmaceutical Physician; Research Funder; Academician (Professor); Guideline Developer; Medical Communications Services; Data Manager; Research in Medical Education; Healthcare Consultant; Patient Advocacy Leader; Physician; Health and Care Guideline Developer.

In Round 2: Data Manager; Medical Education Research and Clinician; Guideline Developer; Administrator; Professor.

In Round 3: Data Manager; Consensus Development Facilitator; Professor; Patient Organization; Guideline Developer.

### Delphi results

**Fig 1:**
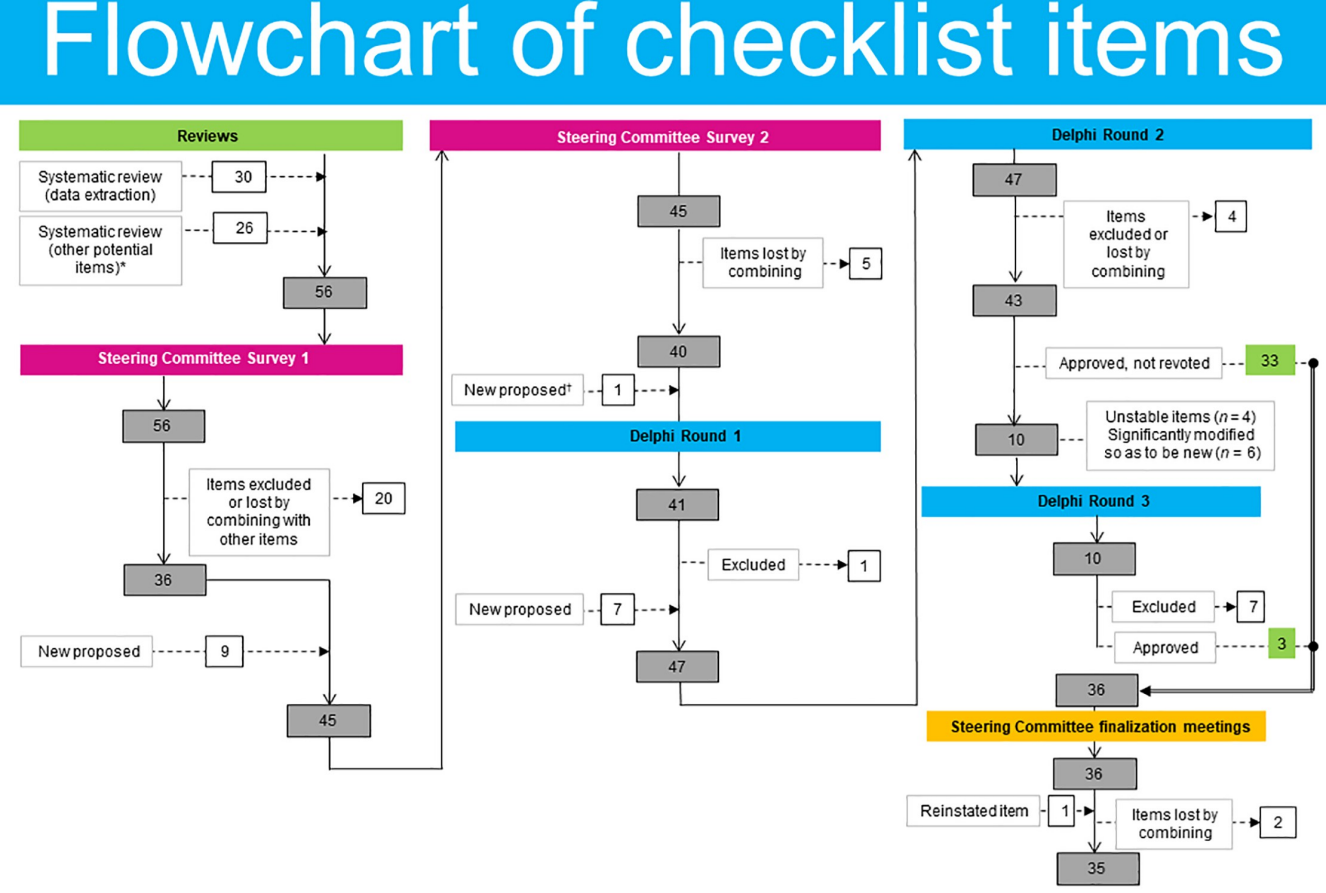
A flow diagram to show the development of checklist items. *Potential items from relevant information beyond the predefined data extraction form [19]. ^†^New item (T1) proposed at checklist review meeting.

The updated preliminary draft checklist presented to the Delphi panel for voting contained 41 items. The changes in the number of checklist items over the Delphi voting rounds are illustrated in Fig 1. After Round 1, 7 new items were added, and 1 item was lost by combining with another item, resulting in 47 items being included in Round 2. Only items that were unstable (*n* = 4) or were modified sufficiently to be considered new (*n* = 6) were voted on in Round 3. After Round 2, 33 items achieved consensus, and a further 3 items achieved consensus after all 3 rounds of voting. Therefore, at the end of the Delphi process, consensus was reached on 36 items. The results of the Delphi process, showing the iteration of items and level of agreement at each round, are summarized in S8 Text.

### Finalization by Steering Committee

One item rejected by the Delphi panel was restored to the checklist (M10, becoming item M5), and 3 highly approved (>90%) items were modified by combining with other items during the Steering Committee finalization workshops. S8 Text contains the iterations of the rounds of the Delphi voting demonstrating the changes made in each round and showing how items evolved.

### Restored item (Delphi M10 > Final M5)

Delphi item M10 (patient and public involvement) failed to achieve stable consensus during the voting process (Round 1, 87.5%; Round 2, 73.1%; Round 3, 76.0%; see S8 Text). The comments from the panel led the Steering Committee to conclude that panelists had not reached agreement on reporting patient and public involvement due to the item being essential in some—but not all—consensus processes (“*Depends on the topic of Delphi consensus*, *should be optional*”; “*For me this rests on the topic of the exercise*”) and because of disagreements about preferred terminology (“*The difference between lay and patient and public partners is potentially confusing*”; “*DO NOT change ‘participants’ to ‘partners’*”). However, the Steering Committee identified many situations where the inclusion of patients would be considered essential. Priority-setting and core outcome identification are just 2 areas where patient participation in consensus exercises is becoming standard [35–37]. Based on unanimous agreement (11/11), the Steering Committee decided to reinstate M10 as reporting item M5, while taking into account the most consistent comments regarding wording (notably, that “lay” should not be used).

### Items with high level of agreement that were modified

Three original items, R3, R6, and R7, overlapped by all covering aspects of which data were reported from the Delphi voting rounds. During the checklist finalization workshops, the Steering Committee discussed these 3 items and combined them to create 2 final items, R3 (quantitative data) and R4 (qualitative data). In addition, the Steering Committee noted an overlap between original items M22 and R8 related to modifications made to items or topics during the consensus process (see S8 Text). These 2 items were combined to create the final item R5. Finally, M13 was revised to remove a conceptual overlap with M12 and to use clearer language.

### Final checklist

The final ACCORD checklist comprised 35 items that were identified as essential to ensure clear and transparent reporting of consensus studies. The finalized ACCORD checklist is presented in Table 4 and is available to download and complete (S9 Text).

**Table 4:** The final ACCORD checklist for the reporting of consensus methods.

Item number	Manuscript section	Item wording	Help text
T1	Title	Identify the article as reporting a consensus exercise and state the consensus methods used in the title.	For example, Delphi or nominal group technique.
I1	Introduction	Explain why a consensus exercise was chosen over other approaches.	n/a
I2	Introduction	State the aim of the consensus exercise, including its intended audience and geographical scope (national, regional, global).	n/a
I3	Introduction	If the consensus exercise is an update of an existing document, state why an update is needed, and provide the citation for the original document.	n/a
M1	Methods > Registration	If the study or study protocol was prospectively registered, state the registration platform and provide a link. If the exercise was not registered, this should be stated.	Recommended to include the date of registration.
M2	Methods > Selection of SC and/or panelists	Describe the role(s) and areas of expertise or experience of those directing the consensus exercise.	For example, whether the project was led by a chair, co-chairs, or a steering committee, and, if so, how they were chosen. List their names, if appropriate, and whether there were any subgroups for individual steps in the process.
M3	Methods > Selection of SC and/or panelists	Explain the criteria for panelist inclusion and the rationale for panelist numbers. State who was responsible for panelist selection.	n/a
M4	Methods > Selection of SC and/or panelists	Describe the recruitment process (how panelists were invited to participate).	Include communication/advertisement method(s) and locations, numbers of invitations sent, and whether there was centralized oversight of invitations or if panelists were asked/allowed to suggest other members of the panel.
M5	Methods > Selection of SC and/or panelists	Describe the role of any members of the public, patients, or carers in the different steps of the study.	n/a
M6	Methods > Preparatory research	Describe how information was obtained prior to generating items or other materials used during the consensus exercise.	This might include a literature review, interviews, surveys, or another process.
M7	Methods > Preparatory research	Describe any systematic literature search in detail, including the search strategy and dates of search or the citation if published already.	Provide the details suggested by the PRISMA reporting guideline and the related PRISMA-Search extension.
M8	Methods > Preparatory research	Describe how any existing scientific evidence was summarized and if this evidence was provided to the panelists.	n/a
M9	Methods > Assessing consensus	Describe the methods used and steps taken to gather panelist input and reach consensus (for example, Delphi, RAND/UCLA, nominal group technique).	If modifications were made to the method in its original form, provide a detailed explanation of how the method was adjusted and why this was necessary for the purpose of your consensus-based study.
M10	Methods > Assessing consensus	Describe how each question or statement was presented and the response options. State whether panelists were able to or required to explain their responses, and whether they could propose new items.	Where possible, present the questionnaire or list of statements as supplementary material.
M11	Methods > Assessing consensus	State the objective of each consensus step.	A step could be a consensus meeting, a discussion or interview session, or a Delphi round.
M12	Methods > Assessing consensus	State the definition of consensus (for example, number, percentage, or categorical rating, such as “agree” or “strongly agree”) and explain the rationale for that definition.	n/a
M13	Methods > Assessing consensus	State whether items that met the prespecified definition of consensus were included in any subsequent voting rounds.	n/a
M14	Methods > Assessing consensus	For each step, describe how responses were collected, and whether responses were collected in a group setting or individually.	n/a
M15	Methods > Assessing consensus	Describe how responses were processed and/or synthesized.	Include qualitative analyses of free-text responses (for example, thematic, content, or cluster analysis) and/or quantitative analytical methods, if used.
M16	Methods > Assessing consensus	Describe any piloting of the study materials and/or survey instruments.	Include how many individuals piloted the study materials, the rationale for the selection of those individuals, any changes made as a result, and whether their responses were used in the calculation of the final consensus. If no pilot was conducted, this should be stated.
M17	Methods > Assessing consensus	If applicable, describe how feedback was provided to panelists at the end of each consensus step or meeting.	State whether feedback was quantitative (for example, approval rates per topic/item) and/or qualitative (for example, comments, or lists of approved items), and whether it was anonymized.
M18	Methods > Assessing consensus	State whether anonymity was planned in the study design. Explain where and to whom it was applied and what methods were used to guarantee anonymity.	n/a
M19	Methods > Assessing consensus	State if the steering committee was involved in the decisions made by the consensus panel.	For example, whether the steering committee or those managing consensus also had voting rights.
M20	Methods > Participation	Describe any incentives used to encourage responses or participation in the consensus process.	For example, whether invitations to participate reiterated, or were participants reimbursed for their time.
M21	Methods > Participation	Describe any adaptations to make the surveys/meetings more accessible.	For example, the languages in which the surveys/meetings were conducted and whether translations or plain language summaries were available.
R1	Results	State when the consensus exercise was conducted. List the date of initiation and the time taken to complete each consensus step, analysis, and any extensions or delays in the analysis.	n/a
R2	Results	Explain any deviations from the study protocol, and why these were necessary.	For example, addition of panel members during the exercise, number of consensus steps, stopping criteria; report the step(s) in which this occurred.
R3	Results	For each step, report quantitative (number of panelists, response rate) and qualitative (relevant sociodemographics) data to describe the participating panelists.	n/a
R4	Results	Report the final outcome of the consensus process as qualitative (for example, aggregated themes from comments) and/or quantitative (for example, summary statistics, score means, medians, and/or ranges) data.	n/a
R5	Results	List any items or topics that were modified or removed during the consensus process. Include why and when in the process they were modified or removed.	n/a
D1	Discussion	Discuss the methodological strengths and limitations of the consensus exercise.	Include factors that may have affected the decisions (for example, response rates, representativeness of the panel, potential for feedback during consensus to bias responses, potential impact of any nonanonymized interactions).
D2	Discussion	Discuss whether the recommendations are consistent with any preexisting literature and, if not, propose reasons why this process may have arrived at alternative conclusions.	n/a
O1	Other information	List any endorsing organizations involved and their role.	n/a
O2	Other information	State any potential conflicts of interests, including among those directing the consensus study and panelists. Describe how conflicts of interest were managed.	n/a
O3	Other information	State any funding received and the role of the funder.	Specify, for example, any funder involvement in the study concept/design, participation in the steering committee, conducting the consensus process, funding of any medical writing support. This could be disclosed in the methods or in the relevant transparency section of the manuscript. Where a funder did not play a role in the process or influence the decisions reached, this should be specified.

UCLA, University of California, Los Angeles.

ACCORD, ACcurate COnsensus Reporting Document; n/a, not applicable; PRISMA, Preferred Reporting Items for Systematic Reviews and Meta-Analyses; SC, Steering Committee.

## Discussion

The ACCORD checklist has been developed using a robust and systematic approach, with input from participants with a variety of areas of expertise, and it is now available for any health researcher to use to report studies that use consensus methods. The process of developing ACCORD itself used consensus methods, which are reported here according to the checklist developed.

### Why ACCORD was needed

The need for optimal reporting of consensus methods has been documented for decades [19,24]. The absence of a reporting guideline that encompasses the range of consensus methods may contribute to poor reporting quality [5], and this prompted the development of the ACCORD checklist.

There are 2 EQUATOR-listed reporting guidelines that provide guidance for specific projects that typically include consensus exercises: AGREE-II has only 1 item, “Formulation of Recommendations,” relating to the method used to obtain consensus [27]. COS-STAR includes only 3 items around the definition of consensus and a “description of how the consensus process was undertaken” [28]. In addition, CREDES [26] is a method- and specialty-specific guideline aimed at supporting the conduct and reporting of Delphi studies in palliative care. None of these guidelines is suitable as a comprehensive and general tool for reporting any type of consensus exercise. ACCORD addresses the breadth of methods used to attain consensus (including the Delphi method) and should be complementary to AGREE-II where a clinical practice guideline also includes a formal consensus development process. Another reporting guideline currently under development, DELPHISTAR [25], is Delphi specific and covers medical and social sciences. ACCORD extends beyond Delphi methods and encompasses a wide range of consensus methods in various health-related fields.

Although familiarity with ACCORD is likely to be useful to ensure relevant elements are considered when designing a consensus study, it is a reporting guideline and not a mandate for study conduct. The methodological background to the items and published examples of what we consider to be good reporting will be discussed in the ACCORD Explanation and Elaboration document (manuscript in preparation).

### Strengths and limitations

ACCORD was conducted through an open, collaborative process with a predefined, published protocol [29]. It started with a systematic review [19] using robust methods of searching, screening, and extraction, which led to the identification of common gaps in reporting consensus methods. Only 18 studies were eligible for inclusion in the systematic review, and data extraction generated 30 potential checklist items. An additional 26 items were identified that were not covered by the data extraction list. Following this thorough process, these 56 potential items were supplemented by a further 9 proposed by the Steering Committee, with an additional 7 proposed by Delphi panelists.

The ACCORD checklist involved input from participants with a wide range of expertise, including methodologists, patient advocates, healthcare professionals, journal editors, publication professionals, and representatives from the pharmaceutical industry and bodies such as NICE and the Scottish Intercollegiate Guidelines Network. With a few exceptions reported here, their recommendations were fully adopted and integrated into the final checklist. ACCORD was developed to assist everyone involved in consensus-based activities or research. It will assure participants that methods will be accurately reported; guide authors when writing up a publication; help journal editors and peer reviewers when assessing a manuscript for publication; and enhance trust in the recommendations made by consensus panels. Our hope is that ACCORD will ultimately benefit patients by improving the transparency and robustness of consensus studies in healthcare.

A limitation of the ACCORD initiative is that the panel was not as diverse as we hoped. ACCORD was a meta-research project drawing on work from many countries, but our view is that diversity of expertise and personal experience always strengthens consensus discussions. Our aim was to broaden the diversity of contributors to ACCORD by recruiting a panel more diverse than the Steering Committee in geography and experience to mitigate the perpetuation of and dilute any biases held by the Steering Committee. Although invitations were sent to potential panelists in South America, Asia, Africa, and Oceania, few responses were obtained, leading to limited participation from these continents and a panel that was largely drawn from Europe and North America. Similarly, the professional diversity of the ACCORD panel was not as broad as we hoped, with patient partners and policymakers relatively underrepresented compared with clinicians. Therefore, in the future, greater efforts should be made to recruit panelists with experience in consensus from a broader range of professions as well as other regions and countries with different cultures and health systems. For example, although some experience of clinical psychology exists in the Steering Committee, inclusion of more behavioral scientists with experience of the process of decision-making could be a helpful addition. Similarly, the inclusion of more policymakers would strengthen the representation of their perspective on consensus reporting to ensure it was relevant and reliable and, therefore, acceptable to be referenced and inform policy. Although these biases were not fully mitigated, future revisions or extensions to ACCORD will aim to improve in this regard.

Members of the ACCORD Steering Committee did not vote in the Delphi surveys. In our process, the virtual workshops held to finalize the ACCORD checklist did not include the Delphi panel. This might be seen as a limitation by some, especially those involved in reporting guidelines development, as a consensus meeting including some expert members of the Delphi panel is usually conducted according to the guidance issued by the EQUATOR Network [32]. However, our process held the Steering Committee and Delphi panel separate: the Steering Committee did not participate in the Delphi panel, and the Delphi panelists did not participate in the final consensus discussions. We suggest that this could in fact be seen as a strength of our process since, while the larger Delphi panel did not reach consensus on 1 particular item, discussion among the Steering Committee led to its inclusion in the final checklist without full approval of the Delphi panel (see results and commentary for item M5). If the panelists had been part of the final consensus meeting, this may have resulted in the omission from the final checklist of this item, which related to patient participation in consensus studies. However, the experience represented by the Steering Committee recognized the value of patient participation in consensus recommendations, the importance of which is reported in the literature [38], and voted to include this item.

Stability of agreement indicates when consensus is present among a group. There are several methods to assess for stability, but ACCORD adhered to a simple definition of achieving the a priori agreed threshold for agreement over a minimum of 2 voting rounds [39].

Another limitation that consensus and survey specialists may note is that the items in our Delphi survey were not presented to panelists in a random order. Since ACCORD was proposing content items for the sections of a scientific manuscript (title, introduction, methods, results, and discussion), we preferred to present items in these sections in the order that they usually appear to enhance comprehension and avoid confusion. This is something that may affect all reporting guidelines development. In fact, several panelists provided feedback on how to order the items.

### The implementation of the ACCORD reporting guideline

Many reporting guidelines are published without initiatives to facilitate implementation. Only 15.7% of guidelines on the EQUATOR Network website mentioned an implementation plan [33]. An implementation study to inform an Explanation and Elaboration document has been completed and the results submitted for presentation at a conference. The full ACCORD implementation plan and supporting materials are being developed and will be available on the ACCORD website (https://www.ismpp.org/accord).

### The future of ACCORD

Robust reporting is particularly important for studies using consensus methods given that so many methods exist and researchers frequently make modifications to “standard” methods. We anticipate that updates of the ACCORD checklist will be necessary, as technology and consensus methods continue to evolve.

Besides updates, ACCORD could have extensions developed in areas such as nonclinical biomedical studies, health economics, or health informatics and artificial intelligence, and even beyond healthcare, with input from appropriate experts. The Steering Committee welcomes feedback and interest from other researchers in these areas.

## Conclusions

The ACCORD reporting guideline provides the scientific community with an important tool to improve the completeness and transparency of reporting of studies that use consensus methods. The ACCORD checklist supports authors in writing manuscripts with sufficient information to enable readers to understand the study’s methods, the study’s results, and the interpretation of those results so that they can draw their own conclusions about the robustness and credibility of the recommendations.

## Supporting information

S1 Text Steering Committee members. (DOCX)

S2 Text Steering Committee surveys. (DOCX)

S3 Text Information pack for Delphi panelist. (DOCX)

S4 Text Example of feedback provided to panelists. (DOCX)

S5 Text Feedback documents provided to Delphi panelists. (PDF)

S6 Text Recommended approaches to approved and rejected items used during the checklist finalization workshops. (DOCX)

S7 Text Criteria for the standardization of terms used to guide reporting in ACCORD. (DOCX)

S8 Text Summary of Delphi voting rounds. (DOCX)

S9 Text ACCORD checklist. (DOCX)
