# Supplementary material for: ACCORD (ACcurate COnsensus Reporting Document): A reporting guideline for consensus methods in biomedicine developed via a modified Delphi
Source: PLoS Med. 2024 Jan 23;21(1):e1004326. doi: 10.1371/journal.pmed.1004326 (PMC10805282; doi:10.1371/journal.pmed.1004326)
Supplement: S1 Text — (DOCX) [file pmed.1004326.s001.docx]

**S1 Text**. The ACCORD Steering Committee responsible for the checklist’s development (alphabetical order, founders in bold)

| **Name** | **Background** | **Country** |
| --- | --- | --- |
| Paul Blazey | Physical therapist, researcher/consensus methodologist, journal editor | Canada |
| **William T. Gattrell** | Medical publications professional working in the pharmaceutical industry | UK |
| **Niall Harrison** | Medical publications professional | UK |
| Ellen L. Hughes | Medical publications professional | UK |
| Amrit Pali Hungin | Medical doctor, professor of primary care and general practice | UK |
| Keith Goldman | Medical publications professional working in the pharmaceutical industry, past practical and educational experience in behavioural sciences (Master’s degree in Clinical Psychology) | USA |
| Patricia Logullo | Postdoctoral meta-researcher with the EQUATOR Network and medical publications professional | UK |
| Amy Price | Research methodologist, journal editor, patient advocate (head and spinal trauma survivor); providing both methodology and lay perspectives | USA |
| David Tovey | Journal editor, medical doctor | UK |
| Christopher C. Winchester | Medical publications professional | UK |
| Esther J. Van Zuuren | Medical doctor, post-doctoral researcher, specialist and consultant in evidence synthesis | The Netherlands |
