# Supplementary material for: ACCORD (ACcurate COnsensus Reporting Document): A reporting guideline for consensus methods in biomedicine developed via a modified Delphi
Source: PLoS Med. 2024 Jan 23;21(1):e1004326. doi: 10.1371/journal.pmed.1004326 (PMC10805282; doi:10.1371/journal.pmed.1004326)
Supplement: S4 Text — (DOCX) [file pmed.1004326.s004.docx]

**S4 Text. Example of feedback provided to panellists. Modifications to the text are underlined**

| **Round 1 statement** M10. Describe the role of any public, lay or patient participants. Detail the stage(s) at which they were involved, and their roles and contributions. Summary and comments from Round 1 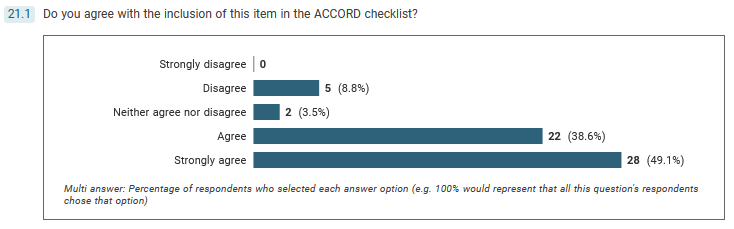   \| *I disagree with the wording "M10. Describe the role of any public, lay, or patient participants." Suggest deleting "lay" and replace with: "M10. Describe the role of public and patient partners."* \| \| --- \| \| *I think rephrase to 'Explicitly detail the stage(s) at which they were involved, and their roles and contributions' - it is really important that the detail is provided, this is key to understanding how stakeholder driven the consensus is* \| \| *M10 no cohort should be singled out. Any participation should be seen as equal by the expertise they bring to the process.* \| \| *It is important to emphasise the patient community voice* \| \| *M 10 Describe the role of any public, lay, or patient participants. Suggest for ALL participants as depending on your question the MDs might be the minority* \| \| *Re 21 and 22. Has the tone and manner of the questions been written to match that of the respondents? (lay language for lay, for example) Have the questions been checked and vetted by the Plain English Society* \| \| *Lay panel members, with expertise anchored in their lived experience, should be accorded as similar a status with professional experts as possible, and language in the checklist should reflect that, Also, should be made clearer that investigators should ensure they describe involvement of lay members of steering committee in earlier methods items* \|  **Modification made between Round 1 and Round 2** Describe the role(s) of any public, lay or patient participants in the different stages of the study. If these groups did not participate, justify why this was the case. |
| --- | --- | --- | --- | --- | --- | --- | --- |
