# Supplementary material for: ACCORD (ACcurate COnsensus Reporting Document): A reporting guideline for consensus methods in biomedicine developed via a modified Delphi
Source: PLoS Med. 2024 Jan 23;21(1):e1004326. doi: 10.1371/journal.pmed.1004326 (PMC10805282; doi:10.1371/journal.pmed.1004326)
Supplement: S6 Text — (DOCX) [file pmed.1004326.s006.docx]

**S6 Text. Recommended approaches to approved and rejected items used during the checklist finalisation workshops**

| **Approved items** | **Rejected items** |
| --- | --- |
| - Keep item as approved (default) - Keep item with wording changes based on panellist comments (requires unanimous Steering Committee approval) - Keep item, but combine with another item or help text where contents appear to relate strongly to one another (requires unanimous Steering Committee approval) | - Confirm item is rejected (default) - Restore item:   Requirements to restore:   1. item already close to acceptance (70%–80% agreement); 2. positive comments with supporting rationale from panellists; and 3. unanimous Steering Committee approval |
