# Supplementary material for: ACCORD (ACcurate COnsensus Reporting Document): A reporting guideline for consensus methods in biomedicine developed via a modified Delphi
Source: PLoS Med. 2024 Jan 23;21(1):e1004326. doi: 10.1371/journal.pmed.1004326 (PMC10805282; doi:10.1371/journal.pmed.1004326)
Supplement: S7 Text — (DOCX) [file pmed.1004326.s007.docx]

### **S7 Text. Criteria for the standardisation of terms used to guide reporting in ACCORD**

| **Verb** | **Type of information** |
| --- | --- |
| Describe | Processes (e.g., recruitment) or broad topics (e.g., areas of expertise) |
| State | Factual information, e.g., conflicts of interest |
| Explain | Methodological choices that may not be immediately transparent, e.g., criteria for panellist inclusion |
| Report | Reserved for (most) results items |
| Discuss | Reserved for discussion items |
